# Tribological Performance of High-Density Polyethylene (HDPE) and Recycled Polyvinyl Butyral (PVB) Blends During Pin-on-Disk Tests

**DOI:** 10.3390/polym17111512

**Published:** 2025-05-29

**Authors:** Scarlette Alejo-Martínez, Ulises Figueroa-López, Andrea Guevara-Morales

**Affiliations:** Tecnologico de Monterrey, Escuela de Ingeniería y Ciencias, Atizapán de Zaragoza 52926, Mexico

**Keywords:** mechanical recycling, recycled polyvinyl butyral, high-density polyethylene, wear, friction coefficient, solid lubricant

## Abstract

High-density polyethylene (HDPE) is a widely used thermoplastic known for its chemical resistance and ease of processing, but it has limited wear performance and moderate mechanical properties. In this study, recycled polyvinyl butyral (rPVB) was incorporated into HDPE at 5, 10, 15, and 20 wt.% to evaluate its effect on tribological performance. Pin-on-disk wear tests were conducted at 12, 15, and 18 N to assess the coefficient of friction (CoF) and wear resistance. Mean CoF values decreased by up to 40% with rPVB addition, with the best performance observed at 15 wt.% rPVB, although some variation was observed across replicates. SEM analysis revealed that rPVB promotes finer debris and transfer film formation, explaining the CoF reduction. However, wear resistance exhibited a complex trend: while rPVB improved adhesion and reduced material loss at lower loads, volume loss increased at higher loads, likely due to rPVB’s lower hardness. Mechanical testing showed an increase in elastic modulus at low rPVB contents due to higher crystallinity, confirmed by DSC; however, tensile strength and impact resistance decreased with rPVB. The results suggest that incorporating 10–15 wt.% of rPVB into HDPE can enhance frictional performance without severely compromising mechanical integrity, offering a sustainable way to valorize rPVB.

## 1. Introduction

The growing concern about sustainability has highlighted the need for recycling plastics, particularly those used in the automotive sector. Unlike commodity plastics, many of the polymers employed in vehicles, such as polyvinyl butyral (PVB), are engineering or high-performance materials with unique characteristics. PVB is predominantly used in laminated glass for automotive windshields, contributing approximately 1.5 to 2 kg of material per vehicle [1,2]. Given the significant volume of end-of-life vehicles worldwide, this represents an immense opportunity to recycle and repurpose PVB into high-value applications.

Despite its excellent mechanical and thermal properties [3], recycled PVB (rPVB)—whether obtained from end-of-life windshields or as residual scrap from production lines—has largely been underutilized or not effectively used [4]. Current applications for this material include its incorporation into flooring and carpet backing [5] and other low-performance products, which fail to exploit its potential as a high-performance polymer. Recent investigations have explored advanced uses for rPVB, including its role as a toughener for polymers such as polyamide [3,4,6,7] and polyvinyl chloride [8]. In these blends, rPVB has demonstrated the ability to enhance impact resistance when appropriately compatibilized. More recently, use of rPVB as a UV-protective textile coating has also been investigated [9], and it was reported that rPVB coating not only improved the tensile strength of the textile materials but also showed a great improvement in its ultraviolet protection factor. Finally, rPVB has been investigated as a solid lubricant for neat and glass-fiber-reinforced polyamide [10,11,12] and polyoxymethylene [13] due to its good adhesion and low shear strength. In those studies, rPVB has significantly reduced the coefficient of friction (CoF) of the blends.

This study focuses on the potential use of rPVB as a solid lubricant in high-density polyethylene (HDPE) blends. In previous studies, a wide range of modifiers have been explored to enhance the wear resistance and tribological behavior of HDPE. These include inorganic nanoparticles such as fumed silica (SiO_2_), titanium nitride (TiN), and halloysite nanotubes (HNTs), which have shown notable improvements when modified with silane coupling agents to enhance interfacial bonding [14]. Graphite nanoplatelets (GNPs), both untreated and organosilane-modified, have demonstrated solid-lubricating effects, achieving significant increases in wear resistance and reductions in friction at low contents [15,16]. Molybdenum disulfide (MoS_2_) has also been incorporated effectively into HDPE, promoting transfer film formation and frictional heat dissipation [17]. Blending with ultra-high-molecular-weight polyethylene (UHMWPE) has similarly been shown to improve abrasion resistance through plastic deformation and fibrillation mechanisms [18].

While these approaches offer significant tribological performance enhancements, there is growing interest in integrating sustainability principles through green tribology. For example, a recent study [19] demonstrated that incorporating recycled silicon particles recovered from wafer cutting waste into a PTFE matrix significantly reduced both the coefficient of friction and specific wear rate, showcasing the potential of recycled industrial materials as effective solid lubricants in polymer composites. Because tribological behavior is known to be highly system-specific, dependent not only on bulk properties but also on surface interactions and operating conditions [20], even if recycled polymers experience some degradation that limits their use in structural applications, they may still perform well in tribological systems. For example, recycled polycarbonate has shown good sliding performance in components like skateboard wheels, while reprocessed HDPE has exhibited wear resistance comparable to that of virgin UHMWPE, even after multiple extrusion cycles [20,21]. These examples highlight the potential of recycled materials in tribological applications and support further investigation into alternative, circular strategies for performance enhancement.

HDPE is a more promising candidate for blending with rPVB compared to previously studied polymers like PA. While PA has excellent mechanical resistance (e.g., higher elastic modulus and ultimate tensile strength), it is thermally dissimilar to rPVB, raising concerns about degradation during processing. Furthermore, rPVB was found to significantly reduce the mechanical properties of PA blends [11], limiting their practical applications. In contrast, HDPE shares greater thermal compatibility with rPVB, potentially enabling better integration and improved material properties.

Additionally, previous work by the authors [22] has demonstrated the biocompatibility and safety of HDPE/rPVB blends in preclinical in vitro models, paving the way for their use in biomedical applications where wear resistance and low friction coefficients are critical. These findings highlight the importance of mechanical and tribological characterization to evaluate the suitability of HDPE/rPVB blends for such applications. The present study aims to address this need by investigating the mechanical and tribological performance of HDPE/rPVB blends, exploring their potential for innovative, high-value uses of rPVB. This research seeks to contribute to a circular economy and reduce the environmental impact of polymer waste while advancing sustainable materials for engineering applications.

## 2. Materials and Methods

### 2.1. Materials

Polymer blends were produced using a commercial high-density polyethylene (HDPE), under the tradename Alathon H5520 from LyondellBasell (Rotterdam, The Netherlands), and recycled polyvinyl butyral (rPVB) donated by Saint-Gobain Mexico (Morelos, Mexico) from its windshield production line. Maleic anhydride (MA) with a molecular weight of 98.06 g/mol from Meyer (Ciudad de Mexico, Mexico) was used as a coupling agent.

### 2.2. Blends Preparation

HDPE/rPVB/MA blends were produced by mixing HDPE with an initial rPVB/MA blend. rPVB/MA blends were prepared by extruding rPVB with 1 wt.% of MA in a Beutelspacher SB-19 single screw extruder at 120 °C and 90 rpm. The extruded material strands were cooled in a water bath and pelletized. Prior to extrusion, the mixtures of rPVB with MA were dried at 60 °C for 24 h. MA was selected for its role as a reactive compatibilizer in the rPVB/MA blend. By reacting with the hydroxyl groups in rPVB, MA introduces polar reactive sites that enhance interfacial adhesion with nonpolar HDPE, improving overall blend compatibility [23,24,25]. Then, HDPE/rPVB/MA blends were prepared by extruding HDPE with 5, 10, 15, and 20 wt.% of the different rPVB/MA blends at temperatures between 140 and 150 °C and 90 rpm. The extruded material strands were cooled in a water bath and pelletized. The mixtures of HDPE/rPVB/MA were also dried in a fan oven at 60 °C for 24 h before extrusion. The obtained blends were extruded one more time (two in total) to promote a good dispersion of the rPVB/MA blend into the HDPE matrix. To maintain an equal thermal history, the same extrusion process was performed twice on the HDPE matrix used as a control. Blends and their compositions are summarized in Table 1.

Specimens for tensile, impact, and wear tests were injection-molded in a Belken SSF500-k5 machine (Belken, Taichung, Taiwan) at an injection temperature of 180 °C and injection and packing pressures of 35–45 MPa and 20–36 MPa, respectively. The mold was kept at room temperature.

### 2.3. Morphology Analysis

Injection-molded wear specimens (Section 2.8) were cryogenically fractured using liquid nitrogen and etched with ethanol for 9 h to selectively dissolve the rPVB phase as described in ref. [6]. This etching step was necessary to evaluate the dispersion of rPVB within the HDPE matrix, as it enables indirect observation of its distribution through the voids remaining in the matrix, which are not visible in unetched fractured surfaces.

### 2.4. Differential Scanning Calorimetry (DSC)

DSC tests were performed using a Shimadzu DSC-60 device (Shimadzu Corporation, Kyoto, Japan). Specimens were first heated from 25 °C to 200 °C at 10 °C/min under nitrogen flow and then kept at 200 °C for 5 min. The specimens were then cooled to 25 °C at the same rate and held at 25 °C for 5 min. Then, the specimens were reheated to 200 °C at the same rate and finally cooled down to room temperature at 20 °C/min.

### 2.5. Melt Flow Index (MFI)

MFI tests were performed on a Dynisco 5000 Melt Flow Indexer (Dynisco, MA, USA) following the ASTM D1238 standard [26] at 190 °C with a 2.16 kg load. The blends were dried in a fan oven at 60 °C for 24 h prior to testing. Three replicate samples were used.

### 2.6. Shore D Hardness

Hardness measurements were performed on a Shore SI-TV desktop device (Shore Instruments & MFG Co., New York, NY, USA) following the ASTM D2240 standard [27] using a 4 kg load. Sanded wear specimens (Section 2.8) were used. Ten measurements per blend were made.

### 2.7. Tensile Tests

Tensile tests were performed in a Shimadzu AG-I equipment (Shimadzu Corporation, Kyoto, Japan) according to the ASTM D638 standard [28]. Type V specimens were used with a gauge length of 7.62 mm, a narrow section width of 3.18 mm, and thickness of 3.30 mm. Tests were performed at room temperature with a crosshead speed of 10 mm/min. Three replicate samples of each blend were used.

### 2.8. Charpy Impact Tests

Impact tests were performed according to the ASTM D6110 standard [29] on an MT 3016 Charpy impact tester (Hung Ta Instrument Co., Taichung, Taiwan) with a hammer arm length of 358 mm and a mass of 2.25 kg. Notched specimens (12.7 mm × 4.2 mm × 125 mm) with a 2.5 mm deep 45° V-groove were tested. Five replicate samples per blend were used.

### 2.9. Wear Tests

Injection-molded disk specimens with a diameter of 38 mm and a thickness of 3 mm were used for wear tests. Before testing, each specimen was sanded until obtaining a roughness of 0.3 ± 0.03 μm. Pin-on-disk wear tests were performed according to the ASTM G99-17 standard [30] in a CSM tribometer (Anton Paar, Peseux, Switzerland) using 6 mm steel balls (AISI 52100); normal loads of 12, 15, and 18 N; a sliding speed of 1 m/s; a 100 mm track radius; and a sliding distance of 1000 m. Three replicates per blend were used.

### 2.10. Scanning Electron Microscopy (SEM)

Cryogenically fractured etched surfaces, wear tracks, and counterpart steel balls were analyzed in a JSM6360LV scanning electron microscope (JEOL Ltd., Tokyo, Japan). Specimens were sputter-coated with a thin layer of gold prior to observation to eliminate charging.

## 3. Results and Discussion

### 3.1. Morphology Analysis

**Figure 1:**
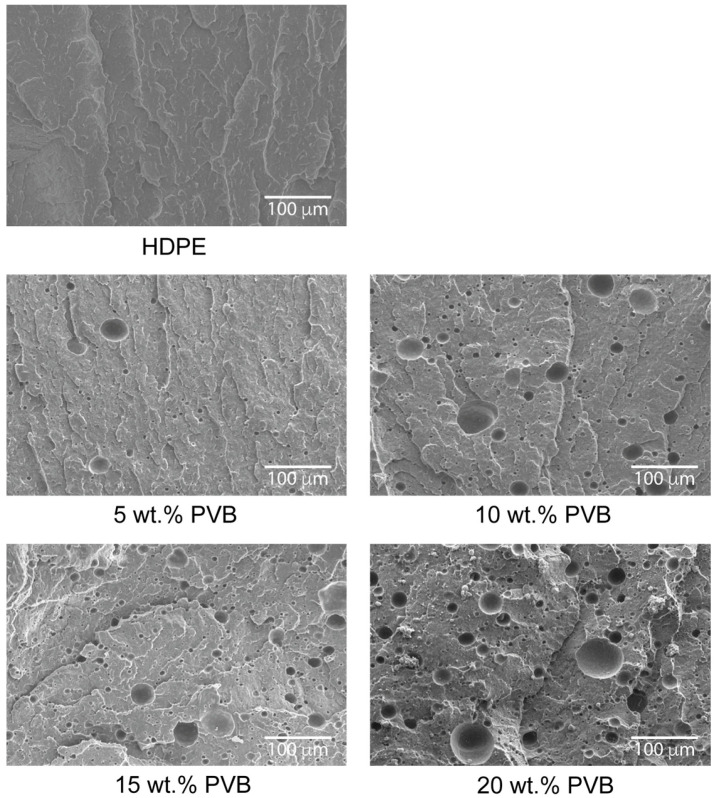
Cryogenically fractured surfaces after etching with ethanol.

In Figure 1, the morphology of the blends is shown. rPVB was dissolved in ethanol, so the remaining voids observed in the micrographs represent the size and dispersion of rPVB particles. Particle size was measured using ImageJ software (version 1.54 g), and the results are summarized in Table 2. As observed, particle size increases with rPVB content, as reported for other rPVB blends [11,13], although for 10 wt.% and 15 wt.%, the size is very similar. Also note that the standard deviation is very high, as the size of rPVB particles varies significantly in every sample.

### 3.2. Dynamic Scanning Calorimetry (DSC)

**Figure 2:**
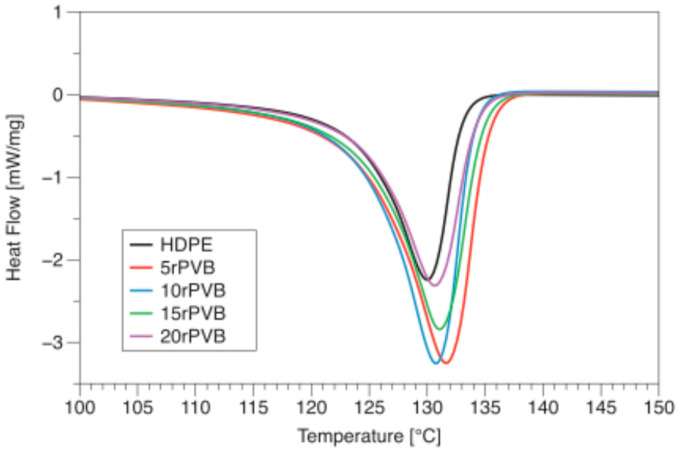
DSC curves of HDPE/PVB/MA blends during the second heating cycle.

The DSC curves of the HDPE/rPVB/MA blends are shown in Figure 2 and summarized in Table 2. The crystallinity of the blends was estimated with Equation (1):

(1) $$X_{c}[\%]=\frac{∆H_{m}}{w_{f}^{PE}∆H_{m}^{0}}\times 100$$

where $∆H_{m}$ is the melting enthalpy of the blends, $∆H_{m}^{0}$ the melting enthalpy of 100% crystallized HDPE (293 J/g [31]), and $w_{f}^{PE}$ the weight fraction of HDPE in the samples.

As observed from Table 2, the addition of rPVB increases the crystallinity of HDPE from 30.7 to 57.5% when 5 wt.% of rPVB is used. This represents an increase of 87% in crystallinity. As rPVB content increases further, crystallinity decreases down to 46.9%. Similar results have been reported for PP [25], in which rPVB particles acted as nucleating agents for crystallization.

### 3.3. Melt Flow Index (MFI)

As observed in Table 2, the addition of 5 wt.% of rPVB slightly increases the MFI of pure HDPE. However, further increases in rPVB linearly decrease (R^2^ = 0.988) the MFI of the blends. For the 20rPVB blend, the MFI decreases down to 16.5 g/10 min, which represents a decrease of 11.8% with respect to HDPE. This agrees with similar works in which rPVB was blended with other polymers such as POM [13] and PA [11,12]. Although rPVB from laminated glasses usually has up to 30 wt.% of plasticizers [3], which may reduce viscosity and thus increase the MFI, rPVB alone typically has a higher molecular weight and lower chain mobility than HDPE. This results in a lower MFI for rPVB (1.71 ± 0.02 and 18.7 ± 0.1 g/10 min for rPVB and HDPE, respectively), which explains the decrease in MFI for blends with 10, 15, and 20 wt.% of rPVB: as the rPVB content increases, the overall viscosity of the blend rises because the polymer matrix is progressively diluted with a component that has less flowability.

### 3.4. Shore D Hardness

The Shore D hardness results are shown in Table 3. Although hardness is slightly lower for blends containing rPVB, the difference is not significant. The values remained between 61.6 ± 1.1 (20 wt.%) and 65.7 ± 0.6 (HDPE), which represents a maximum reduction of 6.2%. Similar results have been reported for PA/rPVB and POM/rPVB blends, in which the hardness of the matrix is usually maintained despite rPVB having a significantly lower hardness (25.6 Shore D [13]) than the matrix (in this case, 65.7 for HDPE according to Table 3). Because Shore D hardness evaluates the resistance of the material to localized deformation, it is primarily determined by the properties of the continuous matrix phase (HDPE), diminishing the effect of rPVB.

### 3.5. Tensile Tests

Elastic modulus (E), ultimate tensile strength (UTS), and strain at UTS results are summarized in Table 3. Contrary to other rPVB blends with PA and POM matrices, the addition of small amounts of rPVB (5–10 wt.%) increases E. Although the standard deviation for the 5rPVB blend is higher than 10%, the results indicate that at 5 and 10 wt.% content of rPVB, the stiffness of the matrix prevails with a certain level of enhancement. It has been reported that PVB has an E of 6.4 MPa [3], whereas HDPE, according to Table 2, has an E = 353 MPa. This does not seem possible according to the rule of mixtures, which predicts an E of 335 MPa for HDPE with 5 wt.% of rPVB. However, this enhancement might be due to rPVB particles acting as nucleating agents for HDPE and thus increasing its crystallinity, as reported in Section 3.2. However, at higher rPVB contents, the effect of rPVB’s low stiffness is more dominant than that of increased crystallinity.

Contrary to E values, UTS values decrease with rPVB content: UTS decreases 6.5% when 5 wt.% of rPVB is used, and 30% when 20 wt.% is added. This decoupling between E and UTS behavior is likely due to the presence of large and non-uniform rPVB particles (as observed in Figure 1), which acted as stress concentrators under tensile loading, and thus promoted earlier failure. This also agrees with the reduction in strain at UTS with rPVB addition. Furthermore, the increase in crystallinity can also contribute to the embrittlement of the blend.

### 3.6. Charpy Impact Tests

Charpy impact resistance decreases with rPVB content as shown in Table 3. For samples with 20 wt.% of rPVB, a reduction of ~47% was found. This is opposite to the toughening effect that has been reported for rPVB blends in other investigations [4,6]. However, in those studies, compatibilizers such as POE-g-MA were used, in which their toughening effect contributes to the toughening of the system. The reduction in impact resistance can be attributed to similar factors to UTS, including poor stress transfer at the HDPE/rPVB interface, the presence of large and irregular rPVB particles acting as stress concentrators, and the increased crystallinity of the matrix that led to a more brittle behavior.

### 3.7. Wear Tests

**Figure 3:**
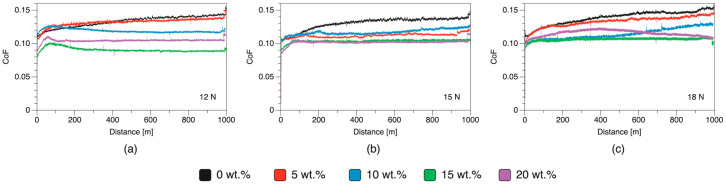
Coefficient of friction (CoF) curves of HDPE/rPVB/MA blends during pin-on-disk wear tests at (**a**) 12, (**b**) 15, and (**c**) 18 N.

The tribological performance of HDPE/rPVB/MA blends was investigated using pin-on-disk wear tests under different loads (12, 15, and 18 N). In Figure 3, CoF curves obtained from the pin-on-disk wear tests are shown. In general, the curves follow the same pattern: there is a sudden increase in CoF due to the application of the load, followed by a running-in period in which the tribological pairs are coupled. It has been reported that at this stage, asperities at the surfaces might be knocked off and as a result, surfaces mate better. After that stage, the CoF stabilizes into a steady-state sliding.

CoF results are summarized in Table 4. As observed, pure HDPE presents the highest CoF at all loads. The addition of rPVB to HDPE reduced the CoF across all tested loads. This reduction was more pronounced at higher rPVB content (20 wt.%), except for blends tested at 12 N and 18 N, where the 15 wt.% PVB blend outperformed others, including samples with 20 wt.% of rPVB. This has been previously reported for PAGF/rPVB and POM/rPVB blends [11,12,13], in which recycled rPVB acts as a solid lubricant, which has been attributed to the formation of a transfer film on the steel counterpart. Although some of the error bars in the CoF results overlap, the mean values show a general decreasing trend with increasing rPVB content. This trend aligns with the expected behavior of rPVB as a solid lubricant and is supported by similar observations in the literature [10,11,13]. However, further statistical analysis (e.g., ANOVA) and additional repetitions could be performed in future work to confirm the significance of these differences and reduce experimental variability.

**Figure 4:**
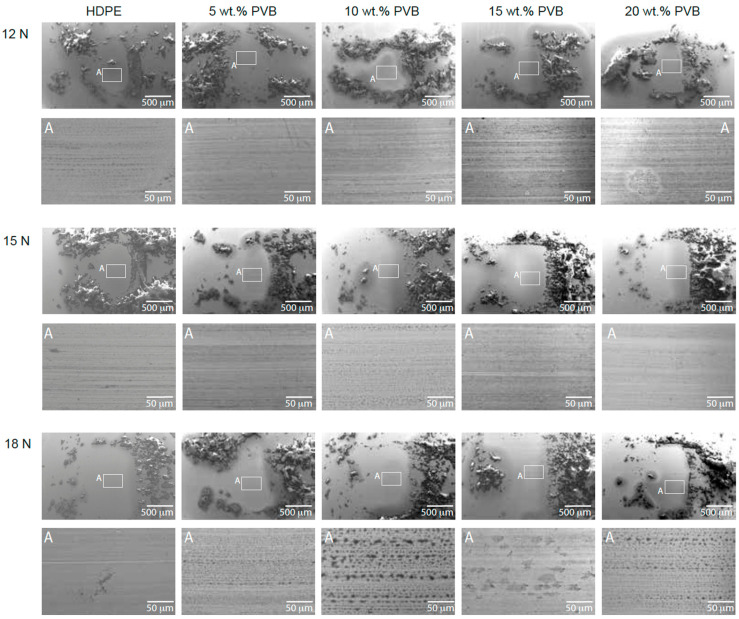
SEM micrographs of steel balls (counterparts) after pin-on-disk wear tests of HDPE/rPVB/MA blends at 12, 15, and 18 N.

In Figure 4, SEM images of the steel balls are shown. Debris is accumulated in all the balls regardless of their composition or load. However, it can be noticed that debris from HDPE samples is bulkier, whereas with rPVB content, the debris becomes finer. Larger debris particles can remain trapped between the contact surfaces, acting as third-body abrasives that increase roughness and therefore the CoF, whereas finer debris particles are less likely to interlock and more prone to act as lubricants, reducing the CoF. Also, finer debris might be acting as third-body rolling elements [32,33], which can decrease friction and increase wear resistance of the polymer matrix acting as micro-bearings. Due to rPVB adhesive properties, it might be harder for large particles to break off from the surface, leading to finer debris and progressive removal, layer by layer, rather than large detachments of material as in the HDPE blend. A closer look into these micrographs is also shown in Figure 4, in which the transfer film located at the middle of the balls is observed in more detail.

At 12 N, the transfer films were uniform across all rPVB contents, supporting the hypothesis that rPVB promotes film retention. For the 15 wt.% sample, the darker color indicates that the material was transferred and accumulated into the valleys of the counterpart, leaving the surface smoother and hence reducing the CoF. No signs of detachment are observed. Similar surfaces are observed at 15 N, although for 10 wt.%, the transfer film appears patchy and non-uniform, indicating that the film was being formed and removed under higher loads. Bahadur [34] concluded that the lack of a continuous transfer film suggests that adhesion between the polymeric transfer film and the steel surface was weak. At higher loads, the wear mechanism might change, so the adhesion effect of rPVB might not be enough to prevent material removal. Also, the transfer film might break down, releasing debris and increasing wear. This is also observed at 18 N (10 and 20 wt.% PVB) and agrees with the higher wear rate of these samples, which will be analyzed next. For the 15 wt.% rPVB blend at 18 N, roll-like debris [34] is observed on the steel counterpart, further suggesting partial removal of the transfer film under higher loads. Note, however, that further characterization of the adhered material using X-ray photoelectron spectroscopy (XPS) or energy-dispersive X-ray spectroscopy (EDX) would be highly valuable to precisely determine the elemental composition and chemical changes occurring at the contact surfaces. Complementary techniques such as confocal Raman microscopy or FTIR could also help to identify organic species present in the transfer film and provide insights into potential tribochemical reactions.

**Figure 5:**
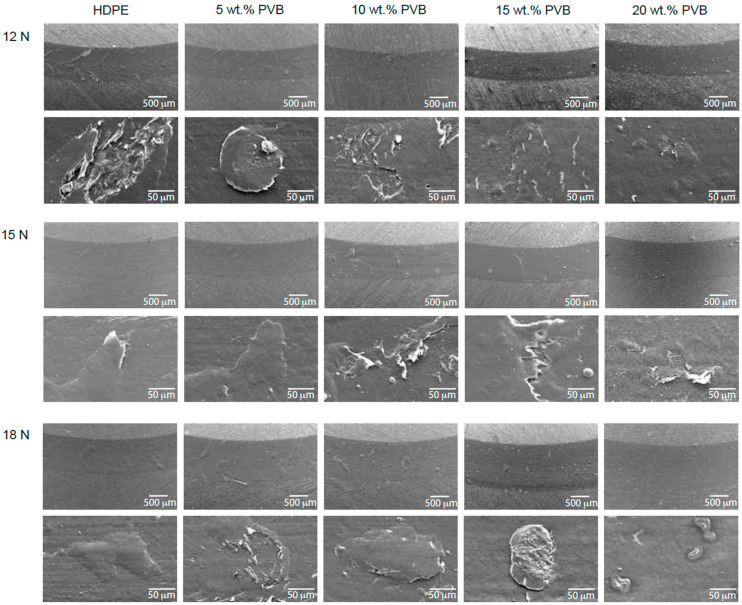
SEM micrographs of the wear tracks after pin-on-disk wear tests of HDPE/rPVB/MA blends at 12, 15, and 18 N.

The resulting wear tracks after the pin-on-disk tests are shown in Figure 5. Their width was measured to estimate the disk volume loss as proposed by the ASTM G99 standard [30] with Equation (2), assuming no significant wear on the steel balls.

(2) $$\mathrm{Disk}\;\mathrm{volume}\;\mathrm{loss}=2\pi R\;[r^{2}-{sin}^{-1}(d/2r)-(d/4)(4r^{2}-d^{2}{)}^{1/2}]$$

where R and d represent the wear track radius and width, and r is the radius of the pin. The results are summarized in Table 4. The volume loss results indicate a complex interaction between rPVB content, applied load, and the competing mechanisms of adhesion and hardness. At 12 N, blends with rPVB exhibited lower volume loss compared to pure HDPE, although no consistent trend was observed across all concentrations. This reduction (up to 16%, or 13% when accounting for experimental variability) suggests that rPVB may initially improve wear behavior by promoting adhesion and transfer film formation, thereby reducing material detachment from the wear track. The authors acknowledge that the relatively modest improvement and overlap in error bars across replicates make it difficult to draw strong conclusions about the magnitude of this effect. Higher magnification SEM images of the wear tracks, shown in Figure 5, support this hypothesis, showing significant detachment of adhered material in pure HDPE and blends with 5 wt.% rPVB, while detachment is progressively minimized in blends with 10, 15, and 20 wt.% rPVB. Note the blister-like features present at 0 and 5 wt.% of rPVB. It seems like material in those regions is not completely adhered and is ready to be detached with further sliding contact. At 15 N, volume loss was very similar for blends with 0 to 15 wt.% of rPVB, but it increased a little bit further for 20 wt.%. At 18 N, however, volume loss increased for all blends containing rPVB. The higher contact force likely overcomes the adhesive effect of rPVB, leading to the removal of the transfer film and greater material detachment from the wear track. SEM images corroborate this, showing that detachment becomes more severe in HDPE/rPVB/MA blends at higher loads. This agrees with [12], in which it was concluded that rPVB initially acts as a binder or adhesive that hinders debris release, decreasing mass loss; however, at higher contact loads, this binding effect is overcome by the lower hardness effect of rPVB, which facilitates material detachment and thus increases volume loss.

## 4. Conclusions

This study investigated the effects of rPVB on the properties of HDPE, focusing on morphological, mechanical, rheological, and tribological behaviors. The incorporation of rPVB led to non-uniform morphologies with increasing particle size at higher concentrations. Melt flow index (MFI) exhibited a decreasing trend with increasing rPVB content, attributed to the higher molecular weight and lower chain mobility of rPVB. Mechanical characterization showed that Shore D hardness remained relatively unchanged across all blends, as this property is mainly governed by the HDPE matrix. Interestingly, the elastic modulus of the blends increased at low rPVB concentrations (5–10 wt.%), a behavior that deviates from other studies in which 5 to 10 wt.% of rPVB decreased the stiffness of PA and POM. DSC analysis indicated that rPVB acted as a nucleating agent, increasing the crystallinity of the HDPE matrix, which explains the stiffening effect. In contrast, both UTS and impact resistance decreased with increasing rPVB content. This reduction in strength and toughness is attributed to the presence of large, non-uniform rPVB particles acting as stress concentrators, as well as the increased crystallinity, which contributes to embrittlement of the matrix, promoting premature failure. Tribological analysis showed that the addition of rPVB led to a general decrease in the CoF of HDPE, particularly at higher rPVB contents. Although some variability was observed among replicates, the mean trend supports the lubricating role of rPVB, attributed to the formation of a more stable transfer film on the steel counterpart and the generation of finer debris, as confirmed by SEM analysis. At lower contact loads, rPVB’s adhesive nature promoted better film retention and minimized wear by preventing large-scale material detachment. However, at higher loads, this beneficial effect was overcome, likely due to the lower hardness of rPVB, leading to increased volume loss. The wear performance of the blends thus exhibited a complex dependence on both rPVB content and applied load: with rPVB acting as a binder at low stresses but contributing to material removal at higher stresses. Although the improvements in tribological performance were moderate and presented variability across replicates, the observed trends are consistent with the expected behavior of rPVB as a solid lubricant. Note, however, that these findings must be weighed against the reduction in mechanical strength, especially for 15–20 wt.%, and the limited effect of rPVB on the thermal transitions of HDPE. Nevertheless, this study contributes to a better understanding of how recycled materials, despite certain trade-offs, can be functionally integrated into tribological systems.

## Figures and Tables

**Table 1:** Composition of HDPE/rPVB/MA blends.

Blend	HDPE (wt.%)	rPVB/MA (wt.%)
HDPE	100	0
5rPVB	95	5
10rPVB	90	10
15rPVB	85	15
20rPVB	80	20

**Table 2:** Results for particle diameter, melt and crystallization temperatures, crystallinity, and melt flow index for HDPE/rPVB/MA blends.

Blend	Particle Diameter [μm]	Tc [°C]	Tm [°C]	Enthalpy (2° Heat) [J/g]	*X_c_* [%]	MFI [g/10 min]
HDPE	-	117.8	130.1	90	30.7	18.7 ± 0.1
5rPVB	4.41 ± 5.47	118.2	131.6	160	57.5	18.9 ± 0.1
10rPVB	8.81 ± 9.82	118.3	130.7	150	56.9	18.2 ± 0.3
15rPVB	8.94 ± 6.15	117.6	131.0	130	52.2	17.1 ± 0.2
20rPVB	11.61 ± 10.48	117.0	130.6	110	46.9	16.5 ± 0.2

**Table 3:** Results for Shore D hardness, tensile, and impact tests for HDPE/rPVB/MA blends.

Blend	Shore D Hardness	Elastic Modulus [MPa]	Ultimate Tensile Strength [MPa]	Strain at UTS [%]	Charpy Impact Resistance [J/m]
HDPE	65.7 ± 0.6	353 ± 29	28.8 ± 3.4	50.6 ± 3.2	66.9 ± 3.7
5rPVB	64.6 ± 1.1	382 ± 48	27.0 ± 2.7	51.1 ± 4.7	49.1 ± 3.2
10rPVB	65.5 ± 1.4	365 ± 13	21.5 ± 0.9	47.4 ± 2.7	45.2 ± 1.2
15rPVB	63.9 ± 1.8	351± 26	21.5 ± 3.3	43.1 ± 3.8	43.2 ± 2.3
20rPVB	61.6 ± 1.1	331 ± 44	20.3 ± 0.2	39.3 ± 1.8	35.7 ± 5.3

**Table 4:** Coefficient of friction and volume loss for HDPE/rPVB/MA blends with 1 wt.% of maleic anhydride (MA) during pin-on-disk wear tests at 12, 15, and 18 N.

Blend	Coefficient of Friction (CoF)			Volume Loss [mm^3^]		
	12 N	15 N	18 N	12 N	15 N	18 N
HDPE	0.140 ± 0.021	0.137 ± 0.008	0.147 ± 0.013	0.812 ± 0.005	0.836 ± 0.005	0.821 ± 0.012
5rPVB	0.135 ± 0.018	0.114 ± 0.01	0.139 ± 0.013	0.713 ± 0.011	0.816 ± 0.007	0.997 ± 0.008
10rPVB	0.117 ± 0.012	0.120 ± 0.017	0.121 ± 0.01	0.677 ± 0.021	0.835 ± 0.016	0.918 ± 0.013
15rPVB	0.089 ± 0.029	0.105 ± 0.023	0.107 ± 0.026	0.693 ± 0.012	0.847 ± 0.003	1.107 ± 0.015
20rPVB	0.105 ± 0.023	0.102 ± 0.028	0.114 ± 0.011	0.735 ± 0.015	0.909 ± 0.008	1.075 ± 0.036

## Data Availability

The raw data supporting the conclusions of this article will be made available by the authors on request.
